# Two-Staged Technology for CoCr Stent Production by SLM

**DOI:** 10.3390/ma17215167

**Published:** 2024-10-23

**Authors:** Polina Kilina, Andrey Drozdov, Alex G. Kuchumov, Evgeniy Morozov, Lyudmila Sirotenko, Andrey Smetkin

**Affiliations:** 1 Department of Innovative Engineering Technologies, Perm National Research Polytechnic University, 614990 Perm, Russia; kilinapn@mail.ru (P.K.); dron.perm@list.ru (A.D.); morozov.laser@gmail.com (E.M.); sirotenko@pstu.ru (L.S.); 2 Biofluids Laboratory, Perm National Research Polytechnic University, 614990 Perm, Russia; 3 Department of Computational Mathematics, Mechanics and Biomechanics, Perm National Research Polytechnic University, 614990 Perm, Russia; 4 Department of Composite Materials Mechanics, Perm National Research Polytechnic University, 614990 Perm, Russia; smetkinaa@pstu.ru

**Keywords:** additive technology, CoCr alloy powder, selective laser melting, metal stent, process–structure–property relationship

## Abstract

Additive manufacturing of porous materials with a specific macrostructure and tunable mechanical properties is a state-of-the-art area of material science. Additive technologies are widely used in industry due to numerous advantages, including automation, reproducibility, and freedom of design. Selective laser melting (SLM) is one of the advanced techniques among 3D fabrication methods. It is widely used to produce various medical implants and devices including stents. It should be noticed that there is a lack of information on its application in stent production. The paper presents the technological aspects of CoCr stent SLM fabrication, including design of stents and development of regimes for their manufacturing. Physical, chemical, and technological properties of CoCr powder were initially determined. Parametric design of mesh stent models was adopted. A two-stage approach was developed to ensure dimensional accuracy and quality of stents. The first stage involves a development of the single-track fusion process. The second stage includes the stent manufacturing according to determined technological regimes. The single-track fusion process was simulated to assign laser synthesis parameters for stent fabrication. Melting bath temperature and laser regimes providing such conditions were determined. Twenty-seven SLM manufacturing regimes were realized. Dependence of single-tracks width and height on the laser power, exposition time, and point distance was revealed. The qualitative characteristics of tracks imitating the geometry of the stent struts as well as favorable and unfavorable fusion regimes were determined. The results of surface roughness regulating of the stents’ structural elements by various methods were analyzed. Thus, this two-staged approach can be considered as a fundamental approach for CoCr stent SLM fabrication.

## 1. Introduction

### 1.1. Clinical Background

Coronary heart disease (CHD) occupies a leading position among cardiovascular diseases of the adult population, mortality, and disability in the world [1,2,3]. Coronary stents are widely used in today’s cardiovascular surgery. They restore blood flow in vessel lumens to reduce complications of CHD [4,5,6,7].

Stents are classified into bare metal stents (BMSs) [8,9], drug-eluting stents (DESs) [10,11], and bioresorbable stents (BRSs) [12,13].

DESs are a type of coronary stent that are coated with a medication, typically an antiproliferative drug, to help prevent the recurrence of blockages (restenosis) after the stent is implanted. While DESs have several advantages over other kinds of stents, they also have some potential disadvantages, including delayed healing and increased risk of thrombosis, potential for hypersensitivity reactions, and cost [1]. The choice between DESs and BMSs should be made on an individual basis, considering the patient’s clinical profile, risk factors, and endovascular surgeon experience [14].

BRSs have a number of potential disadvantages that should be considered when using them. These disadvantages are mechanical strength, duration of biodegradation, inflammatory response, visualization and control, and duration of preclinical testing [15,16]. The thickness of a stent plays an essential role in stent recoil. The polymer alone has a limited mechanical performance and a greater recoil rate. Therefore, a polymeric stent requires thicker struts to achieve similar radial strength in comparison to those relative thinner metallic stents [17]. The polymeric stent might not provide enough radial strength with thinner struts less than 150 μm. On the contrary, thicker struts might lead to increased incidence of in-stent restenosis. Balance between radial strength and in-stent restenosis must be carefully considered. There is still the concern about acute stent recoil and radial strength of bioabsorbable stents because of the different structure between polymeric stents and metallic stents [18]. The above-mentioned limitations slow down the development and implementation of new designs of BRSs into the practice.

The main advantages of BMSs are the shape, flexibility, and lattice structure. The mesh-type geometry of the stent provides a possibility for its optimization to improve short-term and long-term performance in a diseased vessel.

### 1.2. Conventional Methods for Cardiovascular Stent Fabrication

The chosen design and the material to be used determine the stent’s fabrication method, which is knitting, welding, or braiding [19]. Using photochemical etching, electro-forming, and microelectro-discharge machining (μEDM) fabrication techniques, stents without dross can be produced [20]. Cardiovascular stents have been manufactured using laser cutting technology for the past 10 years because of its many benefits, which include fast fabrication speed, high precision, low cost, and dependability [21,22]. Using a direct laser cutting technique with a laser beam, a semi-finished metallic tube ingot is perforated into a mesh-shaped tube to create a stent.

Nevertheless, while these conventional technologies have advantages, they have certain drawbacks as well. For example, simple structures with inadequate radial stiffness for etching or significant issues with material filling and demolding because of the stents’ small size and intricate structure for microelectro-discharge machining [23].

### 1.3. Additive Manufacturing Including SLM Technique for BMS Fabrication

The state-of-the-art technique for fabricating stents—additive manufacturing (AM) or 3D printing—is more cost-effective than laser machining. This technique uses a computer control system to build complicated structures layer by layer. Four distinct categories fall under this approach of stent manufacture: electrospinning (SE), fused filament fabrication (FFF), selective laser sintering (SLS), and selective laser melting (SLM) [24,25,26]. The stent’s structure and design are significantly influenced by the production process, which also has a long-lasting effect on the stent’s microstructure, mechanical qualities, and corrosion behavior. Design of patient-specific mesh-type BMSs by SLM is an actual task in contemporary medicine. SLM has the potential to speed up the product development process by reducing time required for design validation and functional prototyping.

At present, a large number of stent designs have been developed and are in practice, but the question of choosing a rational design and customized production remains open [27,28,29,30,31,32]. The main challenges of SLM stent fabrication are related to accuracy, the quality of the surface layer, and mechanical properties. The requirements of powder composition and its biocompatibility need to be met [33,34,35]. Coronary stents must be rigid enough in the axial direction to resist the compressive force of the artery wall, and at the same time longitudinal flexibility should be enough [36,37]. These characteristics depend on the design of the stent’s mesh and mechanical properties of the stent’s material. Nitinol [38,39,40] and cobalt–chromium alloys [41,42,43] are preferred as starting powders for SLM technology to fabricate biocompatible BMSs. Structures based on these materials have the required set of mechanical, hydrodynamic, and biological characteristics [26,44]. In the SLM process, the laser scans the powder layer by layer, creating the desired geometry, with the powder size used in the SLM process, ranging from 20 to 50 μm, playing an important role in the alloy quality. This size ensures good flowability, bulk density, and shaking density, effective melting and homogeneity of the resulting materials, dimensional accuracy, and high quality of products [45,46].

Experimental stents obtained by the SLM method are mesh structures with weaves ranging in thickness according to a 3D model from 80 to 200 microns [22,47].

Stents with a strut width of 200–250 μm, as well as 130–450 μm, were obtained [48,49].

Defects such as porosity, surface roughness, and the formation of an oxide layer adversely affect the mechanical characteristics of the resulting products [41].

### 1.4. Numerical Modeling of SLM Technique for BMS Fabrication

The requirements of stent quality are extremely high. At the same time, the fabrication method should provide low costs [50]. To accomplish this, the main parameters of SLM printing can be divided into two groups: variable and constant. Variable parameters are fusion regimes. Constant parameters refer to the characteristics of the equipment. At the same time, the most significant parameters are the laser power, the point distance, the exposition time, the scanning speed of the laser beam, the thickness of the powder layer, the size and shape of the melt bath, and a protective atmosphere [51,52].

Numerical simulation is a highly effective method for predicting and assessing the state of complex interconnected physical processes of laser melting. Numerical modeling of the SLM process to manufacture defect-free BMSs with high dimensional accuracy involves application of computational tools and simulation techniques to understand and predict the various physical phenomena involved in the SLM process. Some of the key aspects of numerical modeling in this context include the following: thermal modeling [53], melt bath dynamics [54], microstructure evolution [55], and residual stress and deformation [56].

Determination of temperature fields in the SLM process by direct measurements is very difficult [57]. Mathematical models make it possible to study the influence of technological modes of melting metal powder compositions on the quality of fused products. Defect-free formation of structural elements affects the stability of the SLM process; papers [58,59] present a method for solving the basic thermal equations for laser fusion of a single track. FE models were applied by Li et al. [60], Liu et al. [61], and Khan et al. [62] to estimate temperature profile and predict heat dissipation. It was shown that the melt pool depth increases with layer addition. Following FEM thermal simulation, more complex thermo-mechanical coupling models were introduced to estimate thermal stress level and investigate dependence between scanning pattern and deflection [63,64].

Recent models included neural network application to control the parameters and optimize the power of laser radiation to minimize the energy contribution of the SLM process [65]. The inputs to the model are the laser power, the scanning speed, the degree of overlap, and the scanning strategy. Parameters such as hardness, tensile strength, and porosity were chosen as target functions. It was found that the surface roughness of the fused products increases with the frequency of laser pulses [16]. The laser melting process is characterized by significant temperature gradients in the machining area, which cause the formation of high residual stresses in the finished part [59], leading to a decrease in dimensional accuracy and mechanical characteristics. A numerical model of the temperature distribution near the melt bath in the process of laser synthesis depending on the local geometry of the part [66,67] ensures the stability of the process and the optimization of fusion regimes. To simulate the track fusion process and analyze the thermal distribution, several numerical models have been proposed [57,66,67,68]. The studies showed distributions of the laser beam radiation and temperature fields in the fusion zone. Good recent reviews dealing with modeling of different aspects of SLM fabrication can be found [69,70,71].

### 1.5. Aim of Paper

Despite lot of papers dealing with different aspects of numerical and analytical models for SLM process estimation, there is a lack of papers considering technological aspects of CoCr cardiovascular stent SLM process modeling. Establishing the relationship between the parameters of selective laser melting of CoCr powder and the characteristics of mesh products at different scale levels is a complex task. This research aims to establish the relationship between the laser synthesis parameters of CoCr powder and the quality of stent struts. It should be noticed that there is a lack of information on its application in stent production. The paper presents the technological aspects of CoCr stent SLM fabrication, including raw powder analysis, design of stents and development of regimes for their manufacturing, and CoCr stent surface quality estimation.

## 2. Materials and Methods

### 2.1. Brief Methodology Description and Design of Study

**Figure 1:**
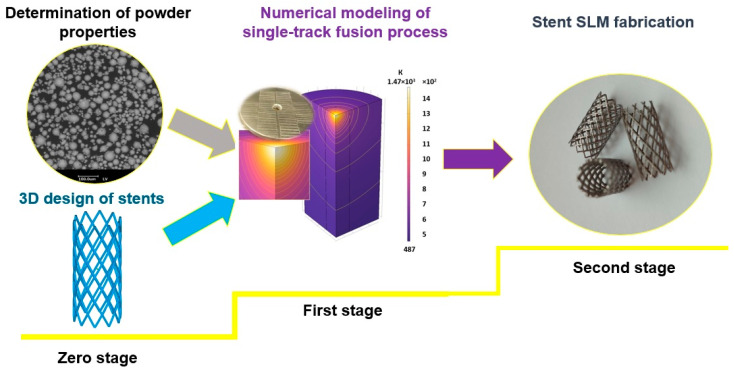
Methodology diagram.

A two-stage approach was developed to ensure dimensional accuracy and quality of stents (Figure 1). The initial (zero) stage assumes design of stents (Section 2.2) and determination of physical, chemical, and technological properties of CoCr powder (Section 2.3). The first stage is numerical modeling of a single-track fusion process in the technological range to determine width, height, depth of penetration, and temperature fields for the selected CoCr powder (Section 2.4). As a result of modeling, the width, height, and depth of fusion of a single width track simulating stent struts are estimated. Minimum values of width and height lead to unacceptable thinning of the bridges. Maximum values lead to large deviations from the 3D model due to the increase in the size of the melt bath. The depth of penetration must be sufficient to ensure the bonding strength of all layers. At the minimum depth of penetration, porosity between the layers is formed, and the maximum depth of penetration leads to additional thermal impact on the underlying layers. As a result of modeling, the interval values for the whole range of modes were estimated, and in situ experiments were carried out on a refined range of values according to the criteria given above (“optimal” width, height, depth of penetration). After that, a regression dependence for track width and height could be obtained. At the second stage, stent SLM fabrication on selected regimes is performed (Section 2.5). Control of macro- and microgeometry should also be performed.

### 2.2. Three-Dimensional Model Preparation and Design Engineering

**Figure 2:**
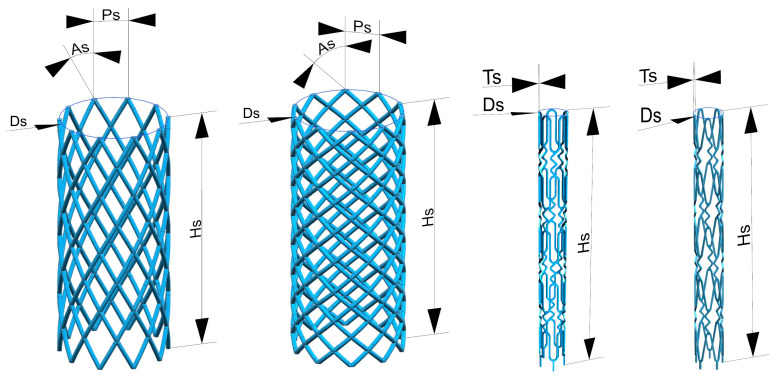
Stent 3D models.

Design of SLM fabricated stents should take into account either patient-specific features [27,28,29,30,31,32] or material properties (like biocompatibility, etc.) [19]. The stents were designed by NX10 (Siemens PLM Software, Munich, Germany). Post-processing of the stl files was performed by utilization of the Magics software package 13 (Materialise, Leuven, Belgium). A parametric approach was adopted. Geometrical parameters were height (Hs), strut spacing (Ps), number of struts, angle of inclination of the helical line (As), strut thickness (Ts), and diameter (Ds) (Figure 2).

The model was designed by multidirectional stretching and rotation of the cross-section of a single element at the base. After that, structural elements were combined into a single mesh frame. The parametric geometry was adopted because of further fabrication by the SLM method. Positioning of 3D models on the substrate was carried out, and supports were built between the fusion platform and the part for its further defect-free removal, as well as to ensure the specified geometry during the construction process. Then, the part was cut into layers according to the layer thickness 20 μm to be processed.

### 2.3. CoCr Powder Testing

CoCr metal powder combines good corrosion and wear resistance according to ASTM F75 [72]. CoCr is one of the most commonly used alloys in medical and dental applications [73]. CoCr alloys are used for surgical implants according to ASTM F1537 [74] and ISO 5832-12:2019 [75]. The main physical, chemical, and technological properties of the CoCr powder were determined. Particle size analysis was carried out in a liquid medium by Low-Angle Laser Light Scattering (LALLS) using the Analysette 22 NanoTec (Fritch GmbH, Idar-Oberstein, Germany) device with a wavelength of 650 nm. A total of 5 measurements of powder were made. The studies were carried out on 5 samples of powder.

The particles shape, their surface structure, and particle size distribution determine properties such as bulk density and shaking density. Density was measured according to the standard methodology ISO 3923-1:2018 [76] (Metallic powders. Determination of apparent density) and ISO 3953:2011 [77] (Metallic powders. Determination of tap density).

CoCr powder particles were examined on an Olympus GX51 Inverted Metallurgical Microscope (Olympus, Tokyo, Japan) with a magnification range of 100–1000×. A COXEM EM-30AXPlus scanning electron microscope (Coxem Co., Ltd., Daejeon, Republic of Korea) combined with an energy dispersive spectrometer with a possible magnification in the range of 20–150,000× and an accelerating voltage of 20 kV was used to obtain images of CoCr powder particles and to facilitate powder element analysis.

### 2.4. Numerical Simulation and Selective Laser Melting of Tracks

The initial data to solve the thermal problem were as follows:

1. Properties of CoCr powder and metal steel substrate.
2. Geometrical characteristics of the substrate with a layer of CoCr powder distributed over the surface.
3. Distribution of laser power over the area of the fusion spot.

CoCr powder’s melting temperature is 1350–1430 °C, tap density is 4.820 g/cm3, and total thermal conductivity is 55.267 W/(m*K). The Heat Transfer module was used to determine the temperature distribution over the powder layer and substrate surface. Regions with natural convection cooling and radiation as well as thermally insulated and neutral zones were specified as boundary conditions. A triangular mesh was adopted.

Table 1 presents the basic thermal conductivity equation and boundary conditions for the heat transfer problem in selective laser melting [78,79]. Notations in Table 1 are as follows: ρ is the density (kg/m^3^); C_p_ is the specific heat capacity at constant pressure (J/kg K); k is the thermal conductivity (W/m^2^ K); T is the temperature (K); t is the time (sec); x and y are the coordinates; Pg is the average laser power density in the form of 1 m^2^; h is the heat transfer coefficient (W/m^2^ K); ε is the emissivity; σ is the Stefan–Boltzmann constant (W/m^2^ K^4^); T_a_ is the ambient temperature (K).

A factor analysis was performed to obtain dependencies between laser powder, exposition time, and point distance for single-track fusion imitating stent strut geometry.

As a result of preliminary exploratory studies, a range of input parameters were selected: laser power P from 37.5 to 42.5 *W* (parameter X1), exposure time *t* from 20 to 60 μs (parameter X2), and distance between points L from 5 to 15 μm (parameter X3) (Table 2). The height and width of the unit track were considered as the objective function. When designing the experiment, all possible combinations of factors were considered at two levels: upper and lower. A planning matrix was drawn up (Table 3) according to papers [80,81]. Levels of factors variation in the experiment: low level (+) and high level (−). The target functions were the height and width of the unit tracks.

### 2.5. SLM Parameters and Settings

Prototypes and parts were manufactured by selective laser fusion using the Realizer SLM-50 unit (Realizer GmbH, Paderborn, Germany). The unit is equipped with an ytterbium fiber laser, the power range is 25–100 W, and the minimum exposure time (illumination of one point) is t = 20 μs. The minimum point distance of illumination is L = 5 μm.

The maximum distance should not exceed the average size of powder particles and is L = 25 μm [45,46]. The fusion took place on a steel substrate in a protective atmosphere of high-purity argon. The layer thickness was 20 μm for an average CoCr particle size of 26 μm.

Specimens were cut from the platform for further metallographic analysis using EcoCut unit (Electronica India Limited, Kolkata, India) with a 0.25 mm BercoCut wire EDM using pure distilled water. To prepare the samples, the LECO PR4X casting press (Leco Corporation, St. Joseph, MI, USA) was used, with the help of which the samples were pressed into Bakelite. Post-processing in the form of tumbling is assumed, possibly in conjunction with ultrasonic processing. Particles with an average size of 200 μm were used. To eliminate dust formation in the working area and increase productivity, the treatment was carried out in an aqueous solution of functional additives. Processing time in on-table vibratory tumblers was 30 min.

## 3. Results

### 3.1. CoCr Powder Analysis

CoCr powder element analysis is shown in Table 4. ASTM F75 regulates the metallic element percentage in the initial powder. The analysis results meet the standard criteria. The ASTM standard range values are presented in Table 4.

The apparent density and tap density are 4.345 g/cm^3^ and 4.820 g/cm^3^, respectively.

**Figure 3:**
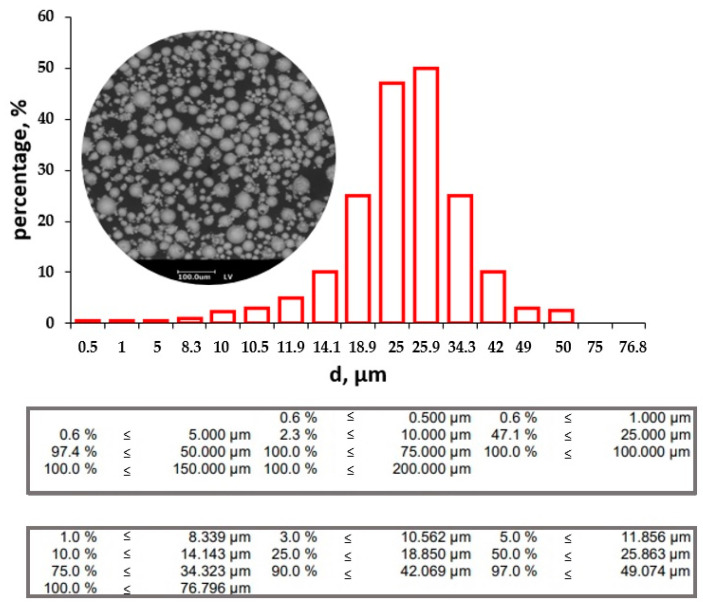
CoCr particle size analysis results.

**Figure 4:**
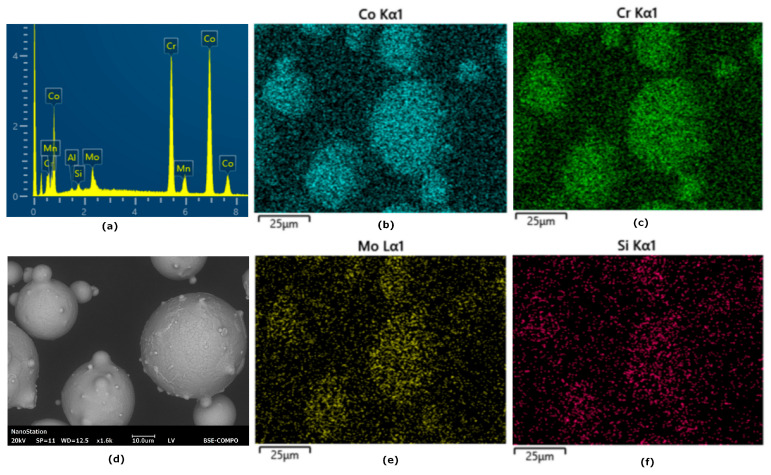
CoCr powder element analysis results (**a**), CoCr powder particles (**b**), Co distribution (**c**), Cr distribution (**d**), Mo distribution (**e**), Si distribution (**f**).

According to the analysis, the CoCr powder particles are spherical with size ranging from 10 to 63 μm. The average particle size is 26 μm (Figure 3). CoCr powder element analysis results are shown in Figure 4. The particle size distribution influences the parameters of the laser melting process, which differ for particles of different dispersion. One of the main characteristics of the powder determined by particle size analysis is the average particle size (d50). Firstly, it largely determines the thickness of a single layer, secondly, the laser power required for processing, and thirdly, the point distance and single tracks. By reducing the particle size, it is possible to increase the degree of elaboration of small structural elements and reduce the roughness of the surface.

The presence of different dispersion particles has a positive effect on the laser melting process, increasing the thermal conductivity of the powder. Thus, at the initial stage, small particles are melted, pores are filled, and large particles are preheated and melted, thereby ensuring a uniform density of the product. In general, the size distribution of particles follows the normal law, which makes it possible to achieve maximum stacking densities.

### 3.2. Numerical Simulation and Single-Track SLM

**Figure 5:**
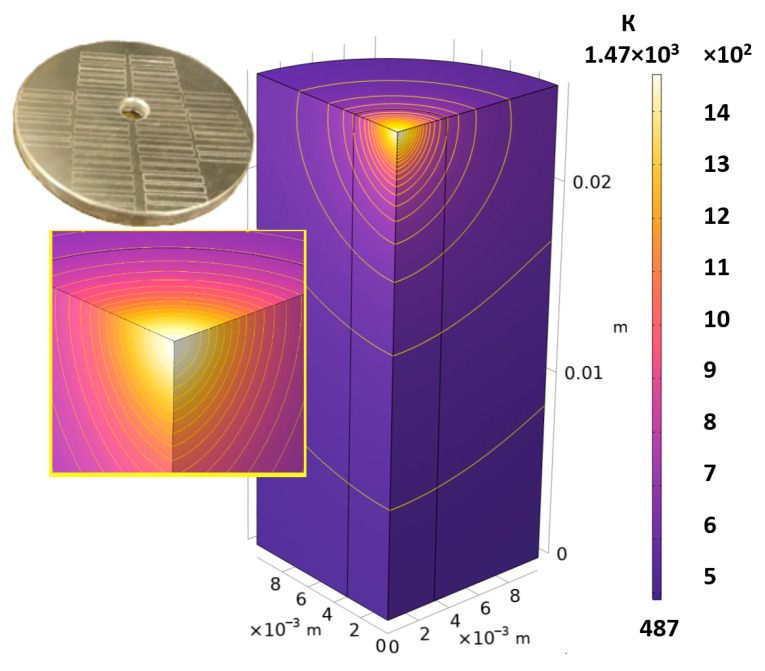
Temperature field distribution diagram in fused single-track contours.

The temperature distribution in the fusion zone was obtained (Figure 5). The temperature distribution range in the fusion zone, obtained as a result of numerical simulation with power variation from 37.5 to 42.5 W, was 1474–2864.73 K. The result of the fusion was a substrate with rectangular contours, represented in Figure 5. In order to provide repetitions for each of the 27 regimes (Table 5), it was decided to fuse the samples in the form of a rectangular outline. In Table 6 and Table 7, a sample of the results of laser fusion of single tracks in the longitudinal and transverse directions is presented.

To validate the model, the predicted width and depth of the melt bath were compared with the actual geometric characteristics of the fused single tracks. The effect of melting regimes on the quality of stent struts was assessed based on the factor analysis results [68,82]. Spatial accuracy is ensured by modeling, as a result of which the required dimensions of the elements of stent structures are determined.

### 3.3. Determination of Width and Height of Fusion Process

**Figure 6:**
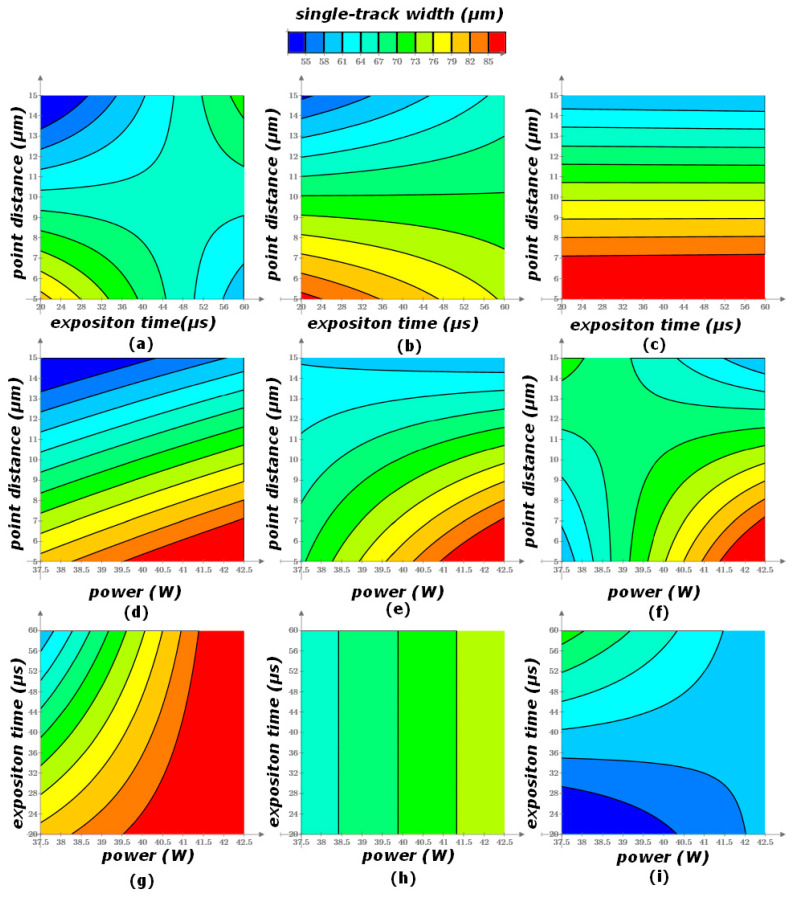
CoCr single-track width: (**a**) P = 37.5 W; (**b**) P = 40 W; (**c**) P = 42.5 W; (**d**) t = 20 μs; (**e**) t = 40 μs; (**f**) t = 60 μs; (**g**) L = 5 μm; (**h**) L = 10 μm; (**i**) L = 15 μm.

**Figure 7:**
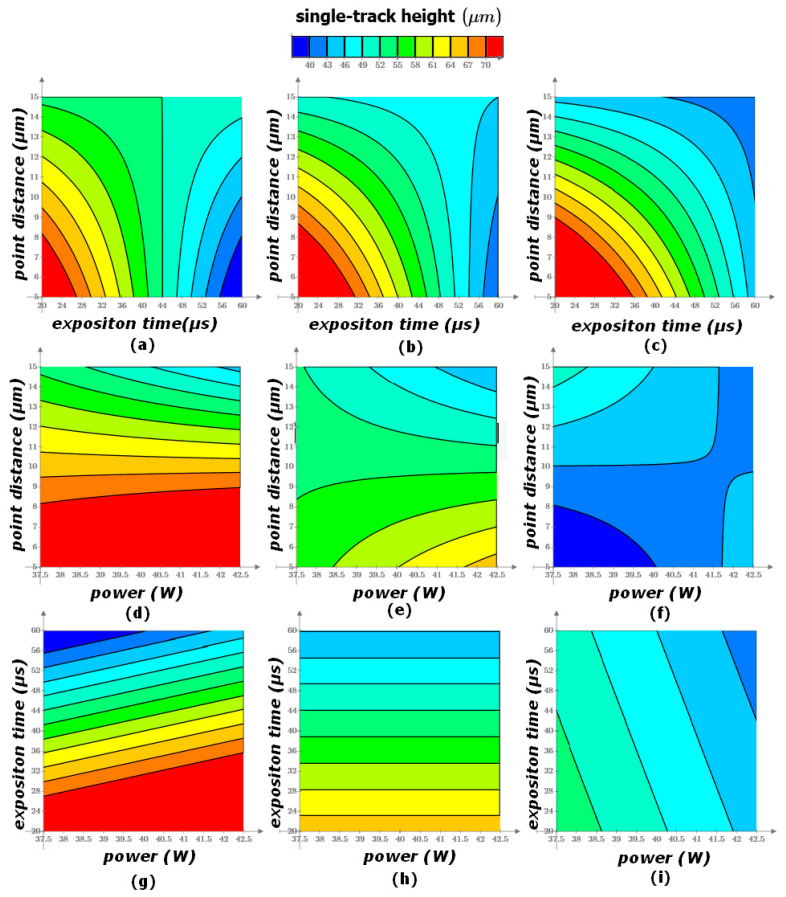
CoCr single-track height: (**a**) P = 37.5 W; (**b**) P = 40 W; (**c**) P = 42.5 W; (**d**) t = 20 μs; (**e**) t = 40 μs; (**f**) t = 60 μs; (**g**) L = 5 μm; (**h**) L = 10 μm; (**i**) L = 15 μm.

As a result of the factor analysis, the single-track width and height were obtained (Figure 6 and Figure 7). Dependences of width and height of the stent struts on the selective laser melting characteristics are described by obtained regression functions:

w = −0.771 × P − 6.962 × t − 10.721 × L + 0.164 × P × t + 0.189 × P × L + 0.464 × t × L − 0.011 × P × t × L + 138.001,(6)

h = 3.670 × P − 1.536 × t + 9.501 × L − 0.367 × P × L + 0.097 × t × L − 17.843,(7)

where P is a laser power (W); t is an exposition time (μs); L is a point distance (μm); w is a single-track width (μm); h is a single-track height (μm).

As the laser power and the point distance increase, an increase in the height of the tracks is observed. However, for the mode with a shutter time of 20 μs and a point distance of 15 μm, the opposite trend is observed, namely, a decrease in the height of the track with an increase in laser power. The reason for the above trends may be that when crossing a certain mark in the value of the point distance (in this case, from 10 to 15 μm), the laser does not sufficiently melt the metal powder, due to the low density of the fusion points.

As the laser power increases, an increase in the width of the tracks is observed (Figure 6a). There are energy input and porosity increases, and boiling of the material occurs. As the exposition time increases, there is a decrease in the width of the tracks for a distance of 5 and 10 μm. For a distance of 15 μm, the width of the tracks is constant as the exposition time increases.

As the laser power increases, there is an increase in the height of the tracks for a distance of 5 μm. For a distance of 10 μm, the height of the tracks is constant as the laser power increases. Finally, for a distance of 15 μm, there is a decrease in the height of the track as the laser power increases. This can be attributed to the fact that with such a gap between the points, the laser cannot fully melt the powder layer.

The result of the analysis of the graphs and the quality of the fused tracks is the diagram presented in Figure 7. In this diagram, the modes in which the high-quality and low-quality tracks were obtained, as well as the high-quality zone, are marked [83,84].

The slower the scanning speed, the longer the laser lingers on the powder, hence the more uniform fusion of the powder. As the scanning speed increases (point distance increases and exposition time decreases), there is a decrease in the geometric parameters of the fusion zone (height and width). If the scanning speed is too slow, the phenomenon of spheroidization and an increase in surface irregularities are observed. As the scanning speed increases, due to the fact that the laser stays on the powder for less time, the geometric characteristics of the fusion zone decrease.

By increasing the laser power, the following trend is observed, namely, an increase in the width of the fusion zone and its depth. This dependence is a consequence of the effect of a higher temperature on the fusion zone, hence an increase in the zone subjected to a higher temperature—an increase in the geometric characteristics of the zone.

The height of the tracks tends to increase at a point distance of 5–10 μm, but after 10 μm it decreases. With an increase in the point distance and the scanning speed, there is a lack of penetration of the track, and a decrease in its height and porosity. The track width increases at the point distance from 5 to 10 μm, and at 15 μm there is a decrease in the width of the fused tracks. As the point distance increases, the laser cannot fully fuse the adjacent dots with each other, which leads to a decrease in the width of the track.

The quality of the tracks was assessed using images taken with an optical microscope. Modes with a large number of pores, cracks, non-penetration of the powder, as well as unfavorable geometry of the track profiles were classified as low-quality.

**Figure 8:**
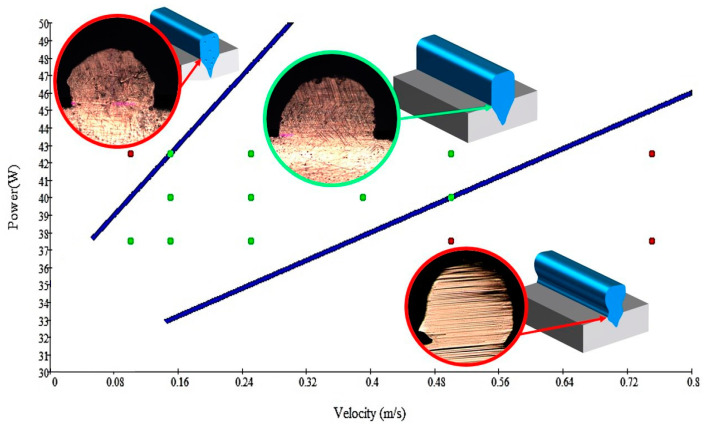
Laser fusion regime map and track profile diagram.

**Figure 9:**
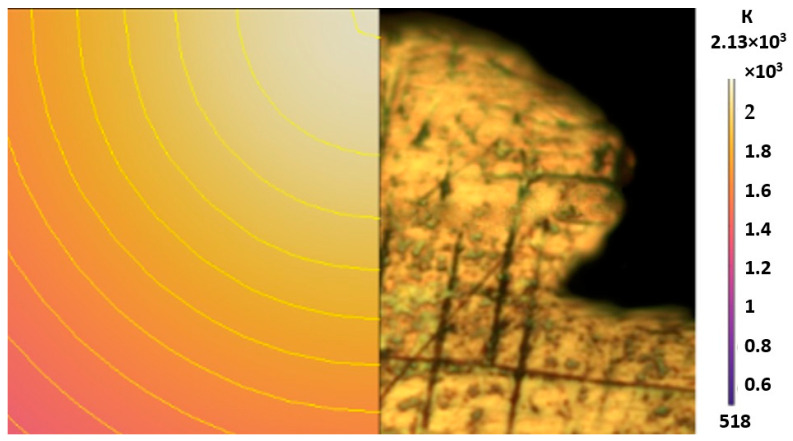
Comparison of the temperature field distribution in the fusion zone in the COMSOL simulation (**left**) and experiment SLM fused single track (**right**).

As a desired profile, profiles with a shape close to rectangular (Figure 8) are considered. With this geometry, the track density is maximized, and the number of gaps and voids between the tracks is minimal. The negative consequences of poor geometry quality are the occurrence of pores, cracks, and residual stresses between the layers of the material. Figure 9 shows the distribution of the temperature fields obtained by computer simulation and the cross-section profile of the track.

From this diagram, you can see that the maximum temperature is reached at the very top of the track profile and there is a gradual decrease as you approach the substrate.

**Figure 10:**
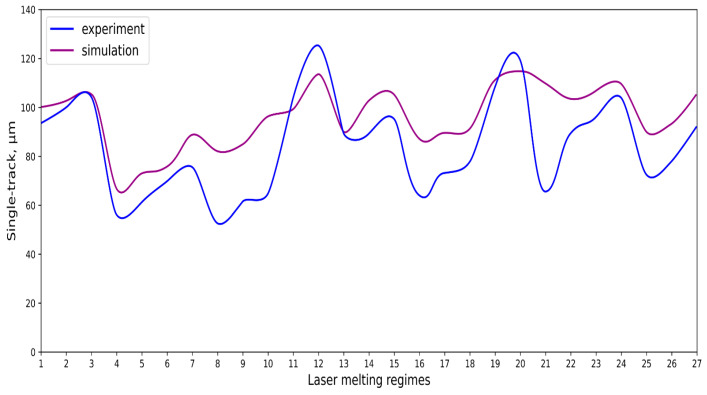
Result comparison graph of simulation and experiment SLM results.

The result of the comparison of the geometric parameters of the tracks, obtained experimentally and with the help of computer modeling, was a graph (Figure 10).

As the power increases, the unit track width increases. The graphs of the experiment and simulation results for the same modes repeat each other. However, the values that stand out from the general picture are noted, since in the process of exposure of the laser beam to the surface of the melt bath, the metal boils. The formation of vapor, microdroplets, and plasma reduces the optical permeability of the gaseous atmosphere and increases the energy dissipation of the laser beam. In this way, the SLM process is self-regulated and the temperature at the surface of the melt bath is reduced. Energy is used to evaporate the material, so the temperature of the melt bath does not exceed the boiling point.

### 3.4. Surface Quality Estimation

**Figure 11:**
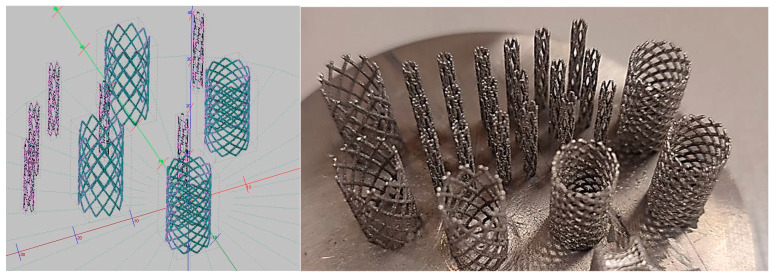
Stents in the slicer program and after the printing.

**Figure 12:**
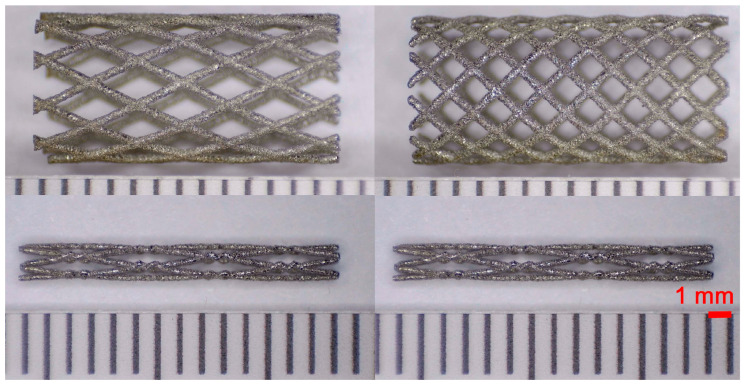
Manufactured CoCr stents.

**Figure 13:**
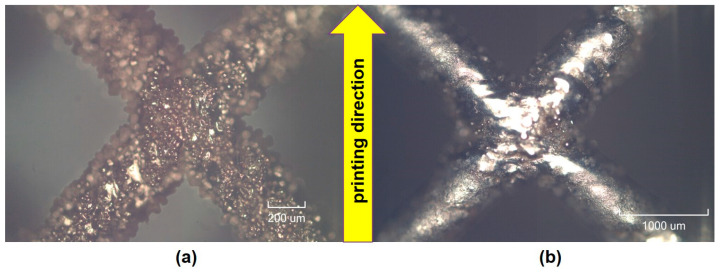
Stent struts (**a**) with spatter particles; (**b**) after ultrasonic tumbling.

As a result of simulation and experimental studies of the melting of CoCr powder-based tracks, simulating the geometry of the mesh material struts, technological modes have been identified that make it possible to form defect-free elements of the frame and are characterized by the formation of stable samples with uniform penetration throughout the volume. In regime No. 17, selective laser melting of CoCr powder was used to obtain mesh structures with different strut widths (Figure 11, Figure 12 and Figure 13).

Based on the studies presented in this article, coronary stent mesh structures with a diameter of 2 to 6 mm with a bridge size of 150 μm to 500 μm were obtained by selective laser melting of CoCr powder. The selected regime range provided the formation of stable samples based on CoCr with an adjustable internal macrostructure. Additional processing methods contribute to changing the microgeometry of the surface depending on the functional purpose of the products.

## 4. Discussion

A two-stage approach was proposed to make more proper geometries of stents. The zero stage assumes design of stents and CoCr powder property evaluation. The first stage is a numerical modeling of the single-track fusion process to determine rational SLM settings for proper strut manufacturing. Numerical simulation assists avoiding numerous experimental studies for determination of optimal regimes for proper production of the stent’s structural elements. At the second stage, SLM fabrication stents on selected regimes is produced.

Despite there being numerous works devoted to CoCr stent SLM printing, all of them do not employ a complex approach related to all stages presented in this paper. One of the crucial parts of this work is the numerical simulation of single-track fusion. Some researchers make numerical simulation to improve SLM process. Nevertheless, this technique was not applied to CoCr SLM stent production yet. Most researchers do not provide SLM printing regimes, which are possible for proper stent production. Here, we employed a technique which assumes three main factors: a laser power, an exposition time, and a point distance. We found the regression dependences for optimal width and height of structural element production.

Also, it can be mentioned that CoCr powder quality should be assessed first, because SLM manufacturing may result in the loss of some alloying components [41]. Structural analysis of produced stent will be studied in the future.

The ability to control the energy input through pulsed wave emission was the primary benefit of the SLM technology that was employed. Unlike continuous linear scans with continuous wave emission, precise positioning of laser pulses in short trajectories is possible with pulsed wave emission. To enhance the surface quality, more processing is necessary for the prototype stents that were manufactured. This suggests that there is still room for improvement in the chemical and electrochemical polishing steps. As was previously noted, melting efficiency can be improved by using the appropriate power and explosion time [41].

The impact of melting regimes on parts manufactured by SLM has been studied computationally and experimentally [85,86]. The findings demonstrated that porosity-free single-track deposition with smaller grains and much greater nanohardness and elongation to failure were achieved by a stable free keyhole melting mode.

A diagram of the temperature field distribution in the fusion zone was constructed, with a variation in power from 37.5 to 42.5 W. The temperature range in the fusion zone was 1474–2864.73 K. The temperature distribution range in the fusion zone obtained as a result of numerical simulation allows complete melting of a single layer of powder, which is the necessary minimum for fusion with the previous layer.

To validate numerical simulation, we performed SLM at 27 different regimes and confirmed a correlation by the metallographic images. The selection of optimal modes (17, 18, 23, 24) (see Table 7) can be recommended. At the same time, the regimes of manufacturing structures with large numbers of pores, cracks, powder non-penetration, as well as incorrect geometry of track profiles were also obtained. These regimes were considered as unfavorable (3, 5, 8).

In the fusion process, the surface roughness is created by spatter particles to the surface (Figure 12), so the initial surface quality of the stent is low. The most promising methods of post-processing are ultrasonic and vibratory tumbling, as well as electrochemical and plasma polishing. Initial roughness after SLM was Rz 25 μm. At the beginning of the treatment, roughness is reduced by removing poorly fixed particles of the powder to be fused. Electrochemical treatment contributed to a reduction in surface roughness by 5–6 times, but its efficiency decreases with a reduction in the sample size, and there are difficulties in the internal surface treatment. It has been found that post-processing ultrasonic treatment makes it possible to treat both external surfaces and internal channels, which have high requirements for surface microgeometry (Figure 13).

The surface quality of the SLM CoCr stent is crucial for fatigue resistance, so surface defects should be avoided. Our results showed that the manufactured stents had a small number of defects due to application of numerical simulation and selection of optimal regimes; nevertheless, we state that the post-production surface process should be conducted for fatigue property improvement [87].

## 5. Conclusions

Fundamental technological foundations for SLM production of CoCr-based stents were developed. We proposed a two-stage approach that includes powder examination, numerical simulation of the SLM process to find optimal regimes for proper strut printing, parametric stent design, SLM manufacturing according to chosen regimes, and post-processing of the stent surface.

Granulometric, morphological, and chemical analysis of CoCr powder was initially performed to determine properties such as bulk and shaking density, particle size, and chemical composition. It was revealed that the powder does not contain harmful impurities and hence it can be used for the manufacture of medical stents.

Parametric stent design makes it possible to vary stent dimensions to control stent shape according to SLM requirements and patient-specific features.

The two-stage approach includes the numerical simulation of single-track width and height dependence on the laser power, exposition time, and distance between points. Thermal distribution of the fusion process was computed. The second stage involved SLM stent manufacturing according to chosen regimes.

To reduce the roughness of the structural element surface after SLM, it is proposed to use ultrasonic treatment. At the same time, it has been established that chemical treatment can significantly reduce roughness, but its efficiency decreases with a decrease in the sample size, and there are difficulties in ensuring the required roughness of the stent’s inner surface. The results obtained are the basis for research and development to reduce surface roughness after SLM. Post-processing modes require further refinement and additional experimentation. Also, one of the priority research directions is biotesting of CoCr materials and stent constructions.

SLM allows the creation of personalized medical structures with an adjustable mesh macrostructure from an individual 3D model. Currently, the task of designing and forming personalized mesh implants from biocompatible materials by selective laser melting is one of the priority areas for the development of the modern medical industry. The results show that SLM can be considered as a substitute operation to microtube manufacturing and laser microcutting for shaping precursors in stent manufacturing. Prototype stents with acceptable geometrical accuracy were achieved and surface quality could be improved through electrochemical treatment and ultrasonic treatment.

We believe that successful application of numerical modeling in the SLM-based fabrication of stents can contribute to the development of more reliable and personalized medical devices, ultimately improving patient outcomes.

## Figures and Tables

**Table 1:** Basic equation and heat transfer model boundary conditions.

Boundary	Boundary Conditions	Equations	No
1	Basic Equation	$\rho C_{p}\left[\frac{\partial T}{\partial t}\right]=k\left[\left(\frac{\partial^{2}T}{\partial x^{2}}\right)+\left(\frac{\partial^{2}T}{\partial y^{2}}\right)\right]$	(1)
2	Heat Flow, Natural Convection Cooling, and Radiation	$-k\frac{\partial T}{\partial x}=P_{g}-h\left(T-T_{a}\right)-\varepsilon \sigma \left(T^{4}-T_{a}^{4}\right)$ 0 µm ≤ X ≤ 50 µm	(2)
3	Natural Convection Cooling, and Radiation	$-k\frac{\partial T}{\partial x}=h\left(T-T_{a}\right)-\varepsilon \sigma \left(T^{4}-T_{a}^{4}\right)$ 50 µm ≤ X ≤ 200 µm	(3)
4	Natural Convection Cooling, and Radiation	$-k\frac{\partial T}{\partial y}=h\left(T-T_{a}\right)-\varepsilon \sigma \left(T^{4}-T_{a}^{4}\right)$ 0 µm ≤ Y ≤ 220 µm	(4)
5	Heat insulation	$\frac{\partial T}{\partial y}=0$	(5)

**Table 2:** Input parameters for 2^3^ factor analysis orthogonal array.

Factors	Coded Notation	Low Level (−)	Main Level (0)	High Level (+)	Step
Laser powder P (W)	X1	37.5	40	42.5	2.5
Exposition time t (μs)	X2	20	40	60	20
Point distance L (μm)	X3	5	10	15	5

**Table 3:** Orthogonal array for 2^3^ factor analysis.

No	X0	X1	X2	X3	X1 X2	X1 X3	X2 X3	X1 X2 X3	y_j1_	y_j2_	y_j3_	$\bar{y_{j}}$
1	+	−	−	−	+	+	+	−	y_11_	y_12_	y_13_	${\bar{y}}_{1}$
2	+	+	−	−	−	−	+	+	y_21_	y_22_	y_23_	${\bar{y}}_{2}$
3	+	-	+	−	−	+	-	+	y_31_	y_32_	y_33_	${\bar{y}}_{3}$
4	+	+	+	−	+	−	−	−	y_41_	y_42_	y_43_	${\bar{y}}_{4}$
5	+	−	−	+	+	−	−	+	y_51_	y_52_	y_53_	${\bar{y}}_{5}$
6	+	+	−	+	−	+	−	−	y_61_	y_62_	y_63_	${\bar{y}}_{6}$
7	+	−	+	+	−	−	+	−	y_71_	y_72_	y_73_	${\bar{y}}_{7}$
8	+	+	+	+	+	+	+	+	y_81_	y_82_	y_83_	${\bar{y}}_{8}$

Levels of factors variation in the experiment: low level (+) and high level (−).

**Table 4:** CoCr powder element analysis.

Chemical Element	Co	Cr	Mo	Si	Mn
Percentage, %	62.16 ± 0.2	30.38 ± 0.2	5.67 ± 0.2	0.75 ± 0.2	0.58 ± 0.2
ASTM F75, %	Balance	27–30	5–7	<1	<1

**Table 5:** Experimental regimes.

No	Exposition Time (μs)	Point Distance (μm)	Laser Power (W)
1	20	5	37.5
2	20	5	40
3	20	5	42.5
4	20	10	37.5
5	20	10	40
6	20	10	42.5
7	20	15	37.5
8	20	15	40
9	20	15	42.5
10	40	5	37.5
11	40	5	40
12	40	5	42.5
13	40	10	37.5
14	40	10	40
15	40	10	42.5
16	40	15	37.5
17	40	15	40
18	40	15	42.5
19	60	5	37.5
20	60	5	40
21	60	5	42.5
22	60	10	37.5
23	60	10	40
24	60	10	42.5
25	60	15	37.5
26	60	15	40
27	60	15	42.5

**Table 6:** CoCr single-track width.

	Laser Power, W		
Mode groups (exposition time, point distance)	37.5	40	42.5
1–3 (20 μs, 5 μm)	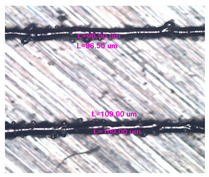	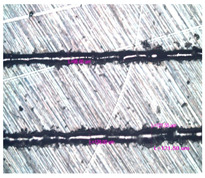	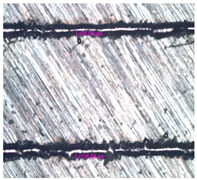
4–6 (20 μs, 10 μm)	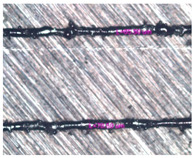	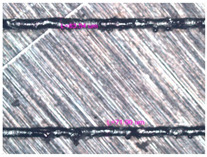	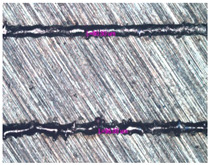
7–9 (20 μs, 15 μm)	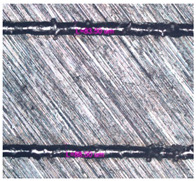	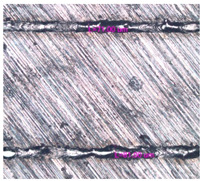	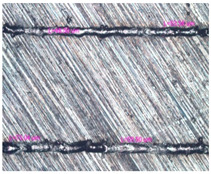
10–12 (40 μs, 5 μm)	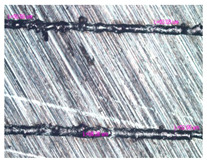	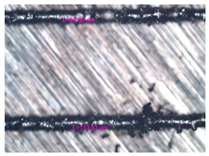	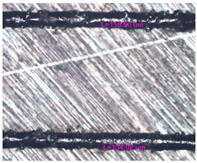
13–15 (40 μs, 10 μm)	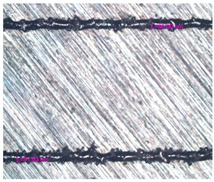	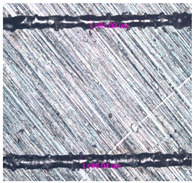	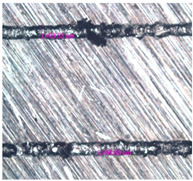
16–18 (40 μs, 15 μm)	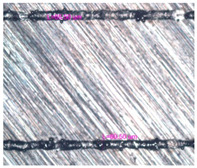	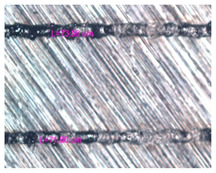	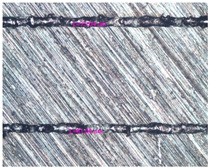
19–21 (60 μs, 5 μm)	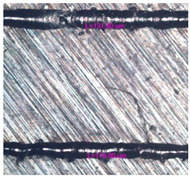	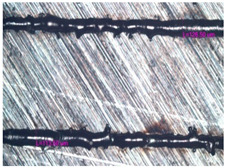	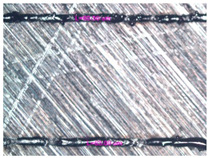
22–24 (60 μs, 10 μm)	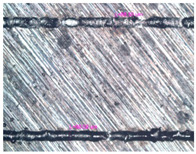	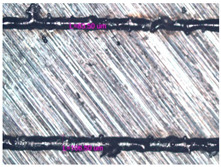	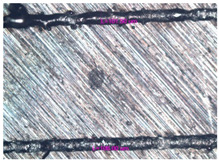
25–27 (60 μs, 15 μm)	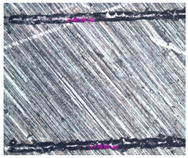	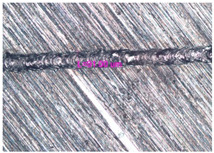	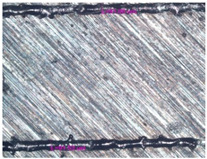

**Table 7:** CoCr single-track height.

	Laser Power, W		
Mode groups (exposition time, point distance)	37.5	40	42.5
1–3 (20 μs, 5 μm)	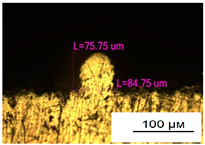	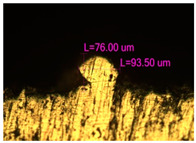	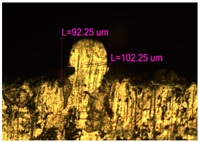
4–6 (20 μs, 10 μm)	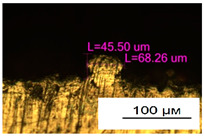	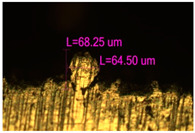	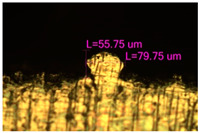
7–9 (20 μs, 15 μm)	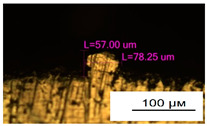	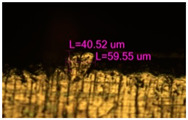	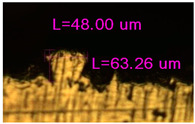
10–12 (40 μs, 5 μm)	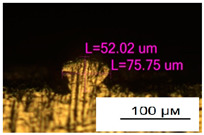	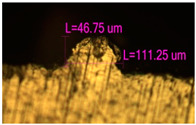	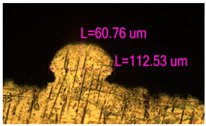
13–15 (40 μs, 10 μm)	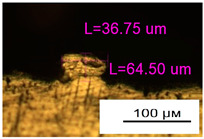	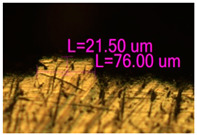	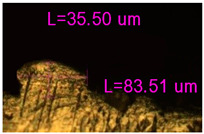
16–18 (40 μs, 15 μm)	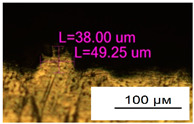	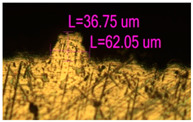	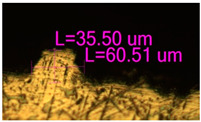
19–21 (60 μs, 5 μm)	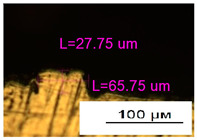	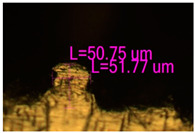	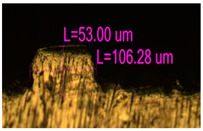
22–24 (60 μs, 10 μm)	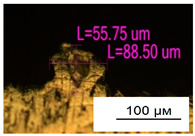	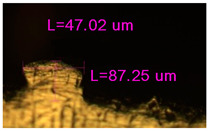	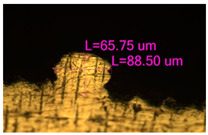
25–27 (60 μs, 15 μm)	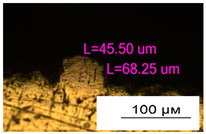	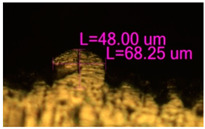	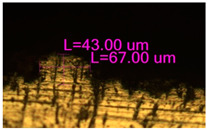

## Data Availability

The original contributions presented in the study are included in the article, further inquiries can be directed to the corresponding author.
